# Supplementary material for: Gender-associated factors for frailty and their impact on hospitalization and mortality among community-dwelling older adults: a cross-sectional population-based study
Source: PeerJ. 2018 Feb 28;6:e4326. doi: 10.7717/peerj.4326 (PMC5834932; doi:10.7717/peerj.4326)
Supplement: Table S1 [file peerj-06-4326-s002.docx]

Supplementary Table 1A. Included and excluded subjects’ demographic and anthropometric characteristics and personal/family medical history

|  | Included (n=1953) | Excluded (n=1126) | p-value |
| --- | --- | --- | --- |
| Demographic features |  |  |  |
| Age, years | 72.72±0.17 | 74.87±0.19 | <0.0001* |
| Race/ethnicity |  |  | <0.0001* |
| Mexican American | 194(3.3) | 125(5.4) |  |
| Non-Hispanic White | 1266(84.7) | 522(72.5) |  |
| Non-Hispanic Black | 297(7.1) | 187(10.9) |  |
| Other Hispanic | 150(2.5) | 84(3.6) |  |
| Other race | 46(2.4) | 57(7.6) |  |
| Marital status |  |  | <0.0001* |
| Married/Living with partner | 1189(64.9) | 445(47.9) |  |
| Widowed/Divorced/Separated | 696(32.4) | 482(48.6) |  |
| Never married | 68(2.7) | 47(3.6) |  |
| BMI**^1,2^** |  |  | <0.0001* |
| Underweight | 25(1.4) | 16(1.7) |  |
| Overweight | 769(38.6) | 303(30.0) |  |
| Obese | 632(31.9) | 370(38.6) |  |
| Normal | 513(27.5) | 216(23.2) |  |
| Family income/poverty ratio | 2.98±0.05 | 2.30±0.06 | <0.0001* |
| Education**^1^** |  |  | <0.0001* |
| Less than 9th grade | 318(8.9) | 258(19.2) |  |
| 9-11th grade | 305(13.6) | 203(18.6) |  |
| High school grad | 495(27.4) | 211(25.8) |  |
| Some college or AA degree | 434(24.9) | 189(23.1) |  |
| College graduate or above | 399(25.1) | 108(13.0) |  |
| Health insurance status |  |  | 0.09 |
| Covered by health insurance | 1900(98.5) | 935(97.4) |  |
| Not covered by health insurance | 53(1.5) | 40(2.6) |  |
| Family/personal medical history |  |  |  |
| Hormone replacement therapy | 406(26.6) | 106(15.3) | <0.0001* |
| Close relative had asthma | 256(13.2) | 121(13.4) | 0.02* |
| Close relative had diabetes | 683(33.8) | 351(36.8) | <0.0001* |
| Close relative had heart attack | 242(14.2) | 127(15.5) | 0.0003* |
| Close relative had osteoporosis | 174(11.0) | 54(8.2) | 0.16 |
| Hospitalization**^1^** |  |  | <0.0001* |
| >3 times | 7(0.4) | 23(2.2) |  |
| <3 times | 322(16.5) | 284(28.4) |  |
| No hospitalization | 1623(83.1) | 667(69.3) |  |
| Mental health consultation | 34(1.8) | 55(5.5) | <0.0001* |
| Osteoporosis**^1^** | 7(0.3) | 14(1.8) | <0.0001* |
| Fracture | 264(14.4) | 152(17.8) | 0.06 |
| Steroid usage**^1^** | 109(6.4) | 83(10.1) | <0.0001* |

1. Summation of percentage is not equal to 100% due to missing data.
2. Four classifications of BMI are defined as: underweight (BMI<18.5 Kg/m2), normal (18.5~24.9 Kg/m2), overweight (25~29.9 Kg/m2), obese (≥30.0 Kg/m2).

Asterisk indicates statistical difference between groups, p < 0.05.
